# Coronary Artery Disease and Aortic Valve Stenosis: A Urine Proteomics Study

**DOI:** 10.3390/ijms232113579

**Published:** 2022-11-05

**Authors:** Luís Perpétuo, António S. Barros, Jéssica Dalsuco, Rita Nogueira-Ferreira, Pedro Resende-Gonçalves, Inês Falcão-Pires, Rita Ferreira, Adelino Leite-Moreira, Fábio Trindade, Rui Vitorino

**Affiliations:** 1 iBiMED—Institute of Biomedicine, Department of Medical Sciences, University of Aveiro, 3810-193 Aveiro, Portugal; 2 Cardiovascular R&D Centre—UnIC@RISE, Department of Surgery and Physiology, Faculty of Medicine of the University of Porto, 4200-319 Porto, Portugal; 3 LAQV/REQUIMTE, Department of Chemistry, University of Aveiro, 3810-193 Aveiro, Portugal

**Keywords:** coronary artery disease, aortic valve stenosis, biomarkers, urine, proteomics

## Abstract

Coronary artery disease (CAD) and the frequently coexisting aortic valve stenosis (AVS) are heart diseases accounting for most cardiac surgeries. These share many risk factors, such as age, diabetes, hypertension, or obesity, and similar pathogenesis, including endothelial disruption, lipid and immune cell infiltration, inflammation, fibrosis, and calcification. Unsuspected CAD and AVS are sometimes detected opportunistically through echocardiography, coronary angiography, and magnetic resonance. Routine biomarkers for early detection of either of these atherosclerotic-rooted conditions would be important to anticipate the diagnosis. With a noninvasive collection, urine is appealing for biomarker assessment. We conducted a shotgun proteomics exploratory analysis of urine from 12 CAD and/or AVS patients and 11 controls to identify putative candidates to differentiate these diseases from healthy subjects. Among the top 20 most dysregulated proteins, TIMP1, MMP2 and vWF stood out, being at least 2.5× increased in patients with CAD/AVS and holding a central position in a network of protein-protein interactions. Moreover, their assessment in an independent cohort (19 CAD/AVS and 10 controls) evidenced strong correlations between urinary TIMP1 and vWF levels and a common cardiovascular risk factor - HDL (r = 0.59, *p* < 0.05, and r = 0.64, *p* < 0.01, respectively).

## 1. Introduction

Cardiovascular diseases (CVDs) are responsible for almost a third of deaths yearly and are expected to reach nearly 24 million cases annually by 2030 [1]. Coronary artery disease (CAD), frequently leading to myocardial infarction, remains the third leading cause of death worldwide [2,3]. Often coexisting with CAD is aortic valve stenosis (AVS), the most common valvular heart disease in the elderly. Over 60% of the patients undergoing surgical or transcatheter valve replacement also have CAD [4]. Moreover, CAD and AVS share similar pathophysiology involving low-density lipoprotein accumulation, oxidation, inflammation, leukocyte infiltration, and multiple risk factors such as hypertension, smoking, age, and dyslipidemia [5,6,7,8]. CAD and AVS are both triggered by a chronic inflammatory process initiated by endothelial dysfunction, which can be caused by stress, genetic factors, chronic infection, or hypercholesterolemia [9,10,11,12,13]. Upon the disruption of the endothelium, LDL particles gradually accumulate and oxidize in the intima layer of the coronaries or inside the fibrosa layer in the case of the aortic valve. This creates a pro-inflammatory environment, which attracts circulating immune cells such as monocytes and T-lymphocytes [14]. The monocytes differentiate into macrophages that recognize and internalize modified lipoproteins but become foam cells with time. The lipid core is released from foam cells, which can occur via necrosis (in CAD) and/or apoptosis (in both CAD and AVS) [15,16]. The apoptotic process contributes to reduced levels/less efficient efferocytosis, removing apoptotic cells from phagocytic cells, resulting in increased tissue necrosis and exacerbated atherosclerosis [17].

AVS and CAD are clinically silent in the early stages of the disease and are often detected in later stages [18]. The AVS and CAD gold-standard diagnostic methods include echocardiography, cardiac computed tomography, and magnetic resonance imaging, in addition to coronary angiography for CAD only [19,20]. Sometimes the diagnosis of CAD is incidental when screening for AVS, and vice-versa, especially in asymptomatic patients. [21,22]. There has been an increasing interest in novel biomarkers to diagnose and predict CAD and AVS in a subclinical stage, as the currently established biomarkers are either non-specific risk biomarkers or reflect myocardial stress/damage, the latest consequence of AVS and CAD. Currently proposed biomarkers include high-sensitivity cardiac troponin (hs-cTn), von Willebrand factor (vWF), IL6, NT-proBNP, or high-sensitivity C-reactive protein (hsCRP) [23,24,25,26,27,28,29].

There is an urge to anticipate CAD/AVS diagnosis to prevent complications and improve patient prognosis. Circulating biomarkers can be important for routine risk assessment, identifying the patients requiring confirmatory diagnostic tests (such as echocardiography or coronary angiography) and, thus, hastening the most appropriate therapeutic regimen. Proteomics can be instrumental in the quest for new biomarkers for these sclerotic conditions [30,31,32]. For a swifter clinical translation, blood proteomics would be tempting. However, with a high dynamic range of protein concentrations up to magnitudes of 10^12^, the typical protein with biomarker value is often masked by high-abundance proteins with limited specificity for the disease [33]. In this sense, urine proteomics can be a viable alternative. The dynamic range of protein concentrations is lower (10^5^–10^6^), as its collection is easier, noninvasive, and is in larger amounts. Furthermore, the proteome is more stable [34,35].

Since CAD and AVS share many risk factors and pathological mechanisms and frequently coexist, the diagnostic workup is often intertwined, and the treatment often coincides (for instance, concomitant aortic valve replacement and coronary artery bypass grafting). Therefore, many of the biomarkers to be identified shall be shared by both conditions. The biomarker-driven suspicion of CAD will require ruling out AVS with confirmatory imaging exams, and vice-versa. Based on this premise, we aimed to characterize the urinary protein profile in patients with CAD and/or AVS, seeking the identification of candidate biomarkers shared by these atherosclerotic-based conditions and envisioning their detection through direct urine analysis in a near future.

## 2. Results

### 2.1. Identification of Potential CAD/AVS Biomarkers through Urine Proteomics

Aiming to identify potentially dysregulated proteins in the urine of patients with atherosclerotic-rooted conditions, we analyzed the proteome of 12 patients (with CAD and/or AVS) and 11 controls (Table 1—Discovery cohort). Sex and age were not significantly different between the two populations. No significant differences were observed in total cholesterol, HDL, LDL, hemoglobin, platelets, and creatinine, but data for the former three were limited. Similarly, no significant differences were found regarding the smoking status and the prevalence of the usual comorbidities such as dyslipidemia, hypertension, or diabetes mellitus. Body mass index was also similar across the cohorts. On the contrary, angina symptoms were only evident among the CAD/AVS patients, and the ejection fraction was significantly reduced in the same cohort.

**Figure 1:**
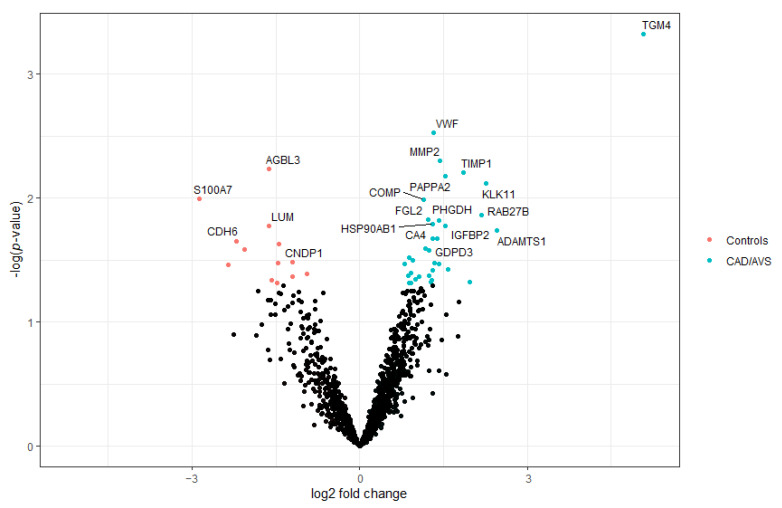
Volcano plot showing the dysregulated proteins in the urine of patients with coronary artery disease or aortic valve stenosis. Blue and red dots mark significantly down- and upregulated proteins in disease. The top 20 significantly changed proteins are labeled with the respective gene name.

**Figure 2:**
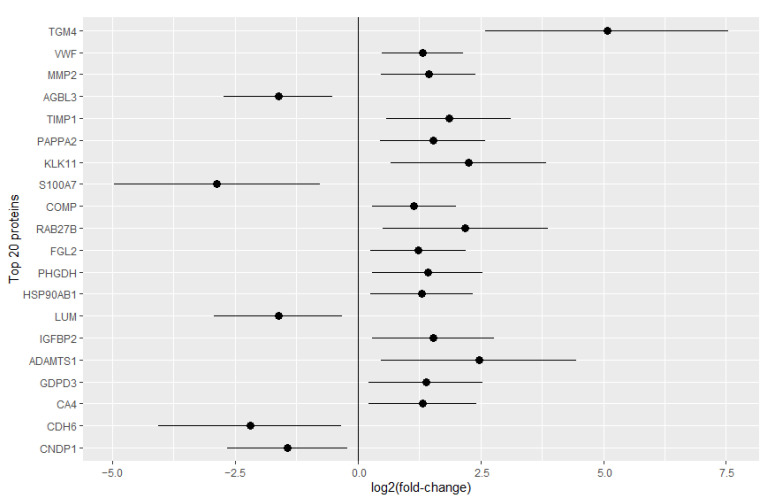
95% confidence intervals of the log2-transformed fold-change of the top 20 proteins showing the most significant changes between cases and controls.

Over 1300 proteins were identified in the 23 samples analyzed from the discovery cohort (Table S1). An exploratory analysis of the identified proteins disclosed 47 that were significantly changed in the urine of CAD/AVS patients (*p* < 0.05) (Figure 1). To identify the best CAD/AVS biomarker candidates, we filtered the top 20 proteins with the most significant changes (Figure 1, labeled dots; Figure 2, depicting the respective log2 fold-change confidence intervals). Among these proteins, the protein-glutamine γ-glutamyltransferase 4 (TGM4) stood out as the protein increasing the most in the urine of diseased subjects. However, TGM4 could not be quantified in 11 samples. Apart from TGM4, the urine levels of other proteins such as the von Willebrand factor (vWF), matrix metalloproteinase-2 (MMP2), metalloproteinase inhibitor 1 (TIMP1), pappalysin-2 (PAPPA2), and kallikrein-11 (KLK11) were found remarkably higher in CAD/AVS (>2.5-fold). Conversely, cytosolic carboxypeptidase 3 (AGBL3) was deemed the most significantly reduced protein in CAD/AVS (3.1-fold).

**Figure 3:**
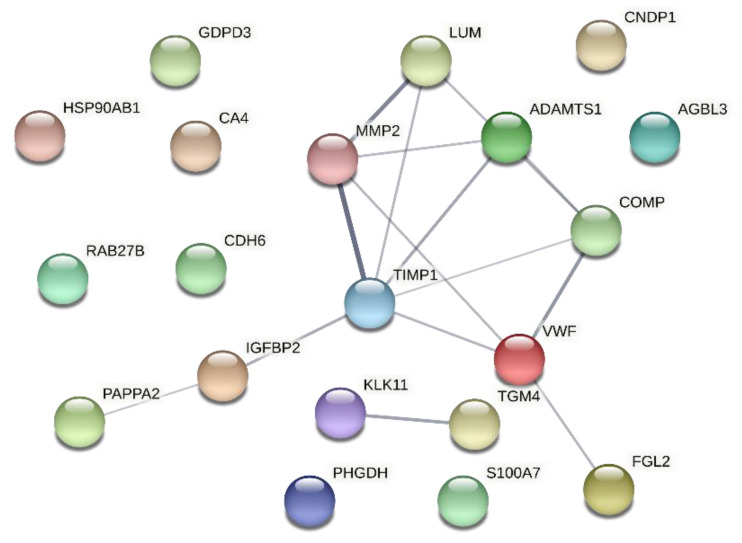
Protein-protein interaction network of the 20 most significantly changed proteins in the urine of patients with coronary artery disease and/or aortic valve stenosis. Edge thickness reflects the confidence score of a given interaction. Proteins are identified with the respective gene name.

With the final goal of selecting the best biomarker candidates for CAD/AVS, we studied the interaction between the Top 20 proteins using STRING to uncover the hub/bottleneck proteins (Figure 3). Network analysis revealed a significant enrichment of protein-protein interactions (*p* < 2.7 × 10^−8^). The proteins TIMP1, MMP2, vWF and A disintegrin and metalloproteinase with thrombospondin motifs 1 (ADAMTS1) popped out as the hub proteins (all having at least four interactions). TIMP1 further showed bottleneck features, dominating the network’s central-most (hub) position. Since TIMP1, MMP2 and vWF occupied the most central proteins and were among the top 5 most significantly dysregulated proteins in the urine of patients with atherosclerotic-rooted conditions, we decided to test these three in an independent cohort of patients by immunoblot.

### 2.2. Slot Blot Relative Quantification of the Putative Biomarkers of Coronary Artery Disease/Aortic Valve Stenosis

To gauge the proteins identified as potential biomarkers to identify patients with atherosclerotic-rooted conditions (CAD/AVS), we compared the urinary levels of TIMP1, MMP2 and vWF in an independent population comprising 29 individuals: 19 CAD/AVS patients and 10 controls (Table 1—Testing cohort). Patients and controls did not show significant differences in total cholesterol or body mass index. Unlike the discovery cohort, however, CAD/AVS patients had higher HDL, LDL, hemoglobin, and lower levels of platelets. Similarly to the discovery cohort, no significant differences were found in smoking habits or in the main comorbidities. Again, angina symptoms were restricted to CAD/AVS patients, and the ejection fraction was reduced in the patients’ cohort.

**Figure 4:**
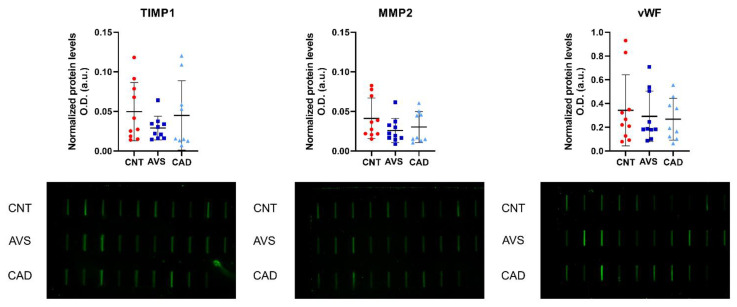
Slot blot relative quantification of TIMP1, MMP2 and vWF in the urine of the controls (red circles), aortic valve stenosis (dark blue squares) and coronary artery disease (light blue triangles) patients from an independent population. Abbreviations: CNT, controls; AVS, aortic valve stenosis; CAD, coronary artery disease.

**Figure 5:**
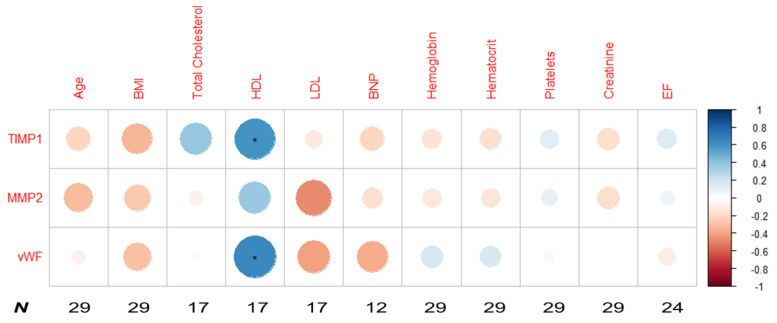
Spearman correlation matrix between the relative urine levels of TIMP1, MMP2 and vWF in the testing cohort and several clinical and biochemical variables. * *p* < 0.05.

The relative quantification of TIMP1, MMP2, and vWF urine levels by slot blot analysis is shown in Figure 4. No significant differences were found in these proteins among the three conditions (CAD, AVS or controls) or between disease (CAD/AVS) and controls. Therefore, we further tested the correlation between the relative levels of TIMP1, MMP2 and vWF (as assessed by slot blot) and several clinical/biochemical variables, and most common cardiovascular disease risk factors: age, BMI, or total cholesterol (Figure 5). Among the tested correlations, we found two positive strong significant correlations between TIMP1 and HDL, and vWF and HDL (r = 0.59, *p* < 0.05, and r = 0.64, *p* < 0.01, respectively).

## 3. Discussion

Given the plethora of similarities between AVS and CAD in pathogenesis, risk factors, and the often-coincidental diagnostic workup, biomarkers for both conditions would be relevant for routine screening and the anticipation of diagnosis. Given the stability and lower dynamic range of urine proteome, we aimed to uncover new candidate markers shared by both conditions through a shotgun proteomic characterization of urine from CAD/AVS patients.

A shotgun approach was applied to profile the urinary proteome of 12 CAD/AVS patients and 11 controls, uncovering close to 50 dysregulated proteins. A closer look at the top 20 dysregulated proteins through protein-protein interaction analysis uncovered vWF, MMP2 and TIMP1 as network hubs/bottlenecks. Therefore, we further analyzed the relative levels of these proteins in an independent cohort and assessed their correlations with particular clinical and biochemical variables.

Our urine proteome analysis pointed to an increase of vWF in the urine of patients with CAD/AVS, suggesting a higher degradation of circulating multimers. The reduction of plasmatic high molecular weight vWF multimers was already associated with AVS [36]. Total plasma levels of vWF in CAD patients were also associated with the severity of coronary stenosis and presented prognostic value for major adverse cardiovascular events [37,38,39]. However, we could not reproduce the findings in an independent cohort. This might be explained by quantifying vWF total levels by immunoblot instead of specific peptides through mass spectrometry (MS). In the future, a deeper analysis of the specific vWF fragments released into urine will be necessary to clarify if the products of vWF degradation bear diagnostic value.

Apart from vWF, our proteomic analysis showed a relative increase of MMP2 and TIMP1 in the urine of patients with CAD/AVS. MMP2, counterbalanced by tissue inhibitors of metalloproteinases (TIMPs), such as TIMP1, are involved in extracellular matrix remodeling and calcification in atherosclerotic plaques and in the aortic valve [40,41,42]. Fitzsimmons et al. previously showed a significant increase in urine TIMP1, but not MMP2, in patients with stable or unstable CAD [43]. Nevertheless, we could not verify the results by immunoblot analysis in an independent population. This might be explained by disease heterogeneity, with patients showing a different number of affected vessels and a different extent of atherosclerotic plaque, adding varying degrees of AVS. Moreover, differences in cohort selection, such as age for the control cohort (Fitzsimmons et al. had an upper limit of 35 years of age for this cohort, while we had no age restrictions, except being over 18 years) and other differences in patient cohort recruitment might explain the differences found between the results of both studies [43].

In spite of the fact that we could not reproduce proteomics findings through immunoblot, we found significant strong positive correlations between TIMP1 or vWF and HDL. Elevated plasma levels of TIMP1 have been associated with the total cholesterol-HDL ratio, suggesting a potential inverse correlation between plasma TIMP1 and HDL [44]. We extend these findings by identifying a direct correlation between urine TIMP1 levels and plasma HDL. The role of urinary TIMP1 as a risk factor for CAD/AVS deserves further scrutiny. Concerning vWF, the initiation of platelet adhesion depends on vWF’s ability to multimerize [45]. However, vWF multimerization may be prevented by HDL, in particular by ApoA-I [45]. The positive association between HDL and vWF suggests a protective role of HDL against the pro-thrombotic effect of vWF, with subsequent secretion of vWF monomers in urine. This will require confirmation in larger studies; however, for now, our results show that urine protein levels of TIMP1 and vWF, respectively, involved in fibrotic remodeling of the atherosclerotic lesions and with thrombosis usually following the rupture of such lesions, are correlated with plasma HDL, an important cardiovascular risk factor.

This study presents some limitations. First, this was an exploratory study with a relatively low number of patients. However, in an attempt to obtain a more homogenous population of CAD/AVS patients, we enrolled all patients on the occasion of the preoperative consultation for either/both coronary artery bypass grafting and aortic valve replacement, which is prioritized for severe cases. Second, this was a retrospective study; some data, including the complete list of prescribed medicines, was missing. Many drugs such as statins or beta-blockers have a disease-modifying effect, which was not appraised in this study. Third, we did not fully control for disease severity or risk factors, which limits the generalizability of the results. These factors may partly explain why we could not replicate MS findings by immunoblot. Alternatively, the differences uncovered by MS might be more relevant at the peptide level, which a standard immunoblot cannot evaluate, but rather by targeted MS approaches.

To the best of our knowledge, this is the first characterization of the urine proteome in patients with CAD and/or AVS that aimed to identify common biomarkers of atherosclerotic conditions. Among the top 20 dysregulated proteins in disease, TIMP1, MMP2, and vWF formed central nodes of a protein-protein interaction network. The lack of replication of the MS findings by immunoblot in an independent cohort might suggest specific differences at the peptide level rather than the intact proteins in the urine. Therefore, future peptide-specific MS-based quantification of urine TIMP1, MMP2 and vWF fragments can be useful in identifying biomarkers for CAD/AVS. Alternatively, the lack of replication can be explained by non-controlled in-patient factors, such as the severity of CAD or AVS. For this reason, in the future, more than matching groups for age, sex, medication and risk factors, parameters such as the number of affected coronary vessels, the degree of coronary or valve stenosis, and the transvalvular pressure gradient must be considered when analyzing urine levels of these proteins/peptides. It will also be important to pursue longitudinal community-based studies to identify the patients with higher risk for disease (through family history or analysis of risk factors like total cholesterol, lipoprotein(a), or body mass index) and compare the urine proteome/peptidome between patients who developed vs. those who did not develop CAD/AVS. Hopefully, this will translate into identifying putative early biomarkers of atherosclerotic-rooted conditions and clinically meaningful correlations, as we have identified for TIMP1 or vWF and HDL, a risk factor for atherosclerotic-related events.

## 4. Materials and Methods

Patients with coronary artery disease and/or aortic valve stenosis were invited to participate in the study on preoperative consultation at the Department of Cardiothoracic Surgery. Recruitment took place between April and November 2018. Patients under 18, pregnant women, patients with active urinary tract infections, and patients with severe mitral or tricuspid insufficiency were excluded. Signed informed consent was obtained from all patients. This study followed the principles stated in the Declaration of Helsinki, and the ethics committee of the Centro Hospitalar Universitário de São João approved the protocol (reference 07-17, 22 May 2017). A total of 31 patients were selected with a diagnosis of CAD/AVS (12 for the discovery cohort and 19 for testing), and 21 controls (11 for the discovery and 10 for the testing cohorts) were recruited on the occasion of a consultation for assessment of cardiovascular risk factors, where, besides such factors, family history, signs and symptoms were investigated to rule out clinically meaningful CAD/AVS. Patients with a clinical indication for myocardial revascularization or aortic valve replacement were not included in this group.

### 4.1. Sample Collection and Preparation

Urine samples were collected before surgical or other therapeutic interventions. Samples were centrifuged at 2000× *g* for 15 min at 4 °C to remove cell fragments and sediments. The supernatants were collected and stored at −80 °C until further processing. Proteins were concentrated by centrifugation at 10,000× *g* for 10 min at 10 °C using a 10 kDa cutoff filter (Vivaspin 500, 10 kDa, Sartorius Biotech, Göttingen, Germany) until saturation of the filter. The protein retentate was resuspended in 100 μL of 0.3 M Tris pH 6.8 and 4% sodium dodecyl sulphate (SDS), and protein concentration was measured using a commercial BCA assay (Pierce, ThermoFisher^TM^, Rockford, IL, USA).

After diluting the whole urine sample, protein concentration was reassessed using the Lunatic UV/Vis polychromatic spectrophotometer (Unchained Labs, Pleasanton, CA, USA). Proteins were treated with 2 mM TCEP(tris(2-carboxyethyl)phosphine) to reduce the disulfide bridges and then with 15 mM iodoacetamide at 30 °C for 30 min for alkylation. A single-pot solid-phase enhanced sample preparation (SP3) method was used for further sample processing. SP3 protein purification, digestion and peptide clean-up were performed using a KingFisher Flex System (ThermoFisher Scientific, Vantaa, Finland) and Carboxylate-Modified Magnetic Particles (GE Life Sciences, MA, USA; GE65152105050250, GE45152105050250) [46]. Beads were conditioned following the manufacturer’s instructions, consisting of three washes with water at a concentration of 1 µg/µL. Samples were diluted with 100% ethanol to a final concentration of 50%. Beads, wash solutions and samples were loaded into 96-well deep-well plates and transferred to the KingFisher. The steps encompassing the collection of beads from the last wash, binding of the proteins to the beads, washing of the beads in solutions 1–3 (80% ethanol), protein digestion (trypsin:protein ratio of 1:50 in 50 mM Triethylammoniumbicarbonat (TEAB), overnight at 37 °C) and the elution of tryptic peptides from the magnetic beads using MilliQ water (Merck Millipore, Darmstadt, Germany) were all automated using the robot. The eluted samples were completely dried to completeness and re-solubilized in 3% acetonitrile, 0.1% formic acid before measurement of peptide content and dilution to a final absorbance of 0.06 at 280 nm.

### 4.2. LC-MS/MS Analysis

A mass spectrometry analysis was performed on an Orbitrap Exploris 480 (Thermo Scientific, Waltham, MA, USA) equipped with a Nanospray Flex Source and coupled to an M-Class UPLC (Waters, Etten-Leur, The Netherlands). Channel A was composed of 0.1% formic acid, and channel B was composed of 0.1% formic acid and 99.9% acetonitrile. For each sample, 5 μL of peptides were loaded on a commercial MZ Symmetry C18 Trap Column (100 Å, 5 µm, 180 µm × 20 mm, Waters, Etten-Leur, the Netherlands) followed by a nanoEase MZ C18 HSS T3 Column (100 Å, 1.8 µm, 75 µm × 250 mm, Waters, Etten-Leur, the Netherlands). A flow rate of 300 nL/min was defined for peptide elution. The gradient was defined as follows: 5% B for 3 min, followed by a gradient from 5 to 35% B in 90 min to separate peptides, 95% B for 5 min to wash the column, and 5% B for 10 min to re-equilibrate the column to starting conditions.

Samples were analyzed in random order. The mass spectrometer was operated in data-dependent mode (DDA) with a maximum cycle time of 3 s, using Xcalibur (Tune version 2.0, Thermo Electron Corporation, West Palm Beach, FL, USA), with spray voltage set to 2.0 kV, funnel RF level at 40%, and heated capillary temperature at 275 °C. Full-scan MS spectra (300−1200 *m*/*z*) were acquired at a resolution of 120,000 at 200 *m*/*z* after accumulation to a target value of 3,000,000 or for a maximum injection time of 45 ms. MS2 spectra were acquired at a resolution of 45,000 after fragmentation with an NCE of 30% using a maximum injection time set to auto and an AGC target of 100,000. Precursors were selected for MS/MS if the intensity reached at least 5000. A filter was applied to only select species with charge states between 2 and 5. Precursor masses previously chosen for MS/MS measurement were excluded from further selection for 20 s, and the exclusion window was set at 10 ppm. The samples were acquired using internal lock mass calibration on *m*/*z* 445.1200.

The mass spectrometry proteomics data were handled using the local laboratory information management system (LIMS) [47], and all relevant data have been deposited to the ProteomeXchange Consortium via the PRIDE (http://www.ebi.ac.uk/pride accessed on 20 October 2022) partner repository with the data set identifier PXD036800.

### 4.3. Data Analysis

MS data analysis was performed with MaxQuant (version 1.6.2.3, Computational Systems Biology, Martinsried, Germany), using the Andromeda search engine for protein identification [48]. Spectra were searched against a Homo sapiens database from the Uniprot reference proteome (taxonomy 9606, accessed on 9 July 2019), concatenated to its reversed decoyed fasta database and common protein contaminants. Carbamidomethylation of cysteine was set as a fixed modification, while variable modifications included methionine oxidation and N-terminal protein acetylation. Enzyme specificity was set to trypsin/P, allowing a minimal peptide length of 7 amino acids and no more than two missed cleavages. MaxQuant Orbitrap default search settings were used. A maximum false discovery rate (FDR) was set to 0.01 and 0.05 for peptides and proteins, respectively. Label-free quantification was enabled, and a 2-min window for a match between runs was applied. Protein fold-changes were computed based on Intensity values based on the MaxLFQ algorithm. To filter for proteins with two or more peptides, allowing no more than five missing values per comparison, to normalize the data using a modified robust z-score transformation and to compute *p*-values using the t-test with pooled variance, we used the R package SRMService functions [49]. If all protein measurements were missing in one of the conditions, data were imputed using a fold-change computed by the mean of 10% smallest protein intensities.

### 4.4. Bioinformatics Analysis

To prioritize the targets for testing, we restrained our analysis to the top 20 most significantly dysregulated urine proteins. To select the best candidates, we gauge how these proteins interact amongst themselves to identify relevant network hubs/bottlenecks. The interactions between the most significantly dysregulated urine proteins were studied using STRING database (v11.5, accessed on 4 April 2022). A score of 0.4 was defined as the minimum threshold regarding protein-protein interactions (medium confidence).

### 4.5. Slot Blot Relative Quantification of Urine Proteins

Aiming to confirm the dysregulation of specific proteins in the urine of CAD/AVS patients, a slot blot analysis was performed using urine samples from an independent group of patients (CAD N = 9, AVS N = 10) and additional control samples (N = 10). Essentially, 40 µg of protein diluted in TBS buffer (10 mM Tris(hydroxymethyl)aminomethane, 150 mM NaCl, pH 8.0) was loaded into the wells in a slot blot apparatus. Protein was transferred with vacuum onto 0.45 µm nitrocellulose membranes (Amersham^TM^ Protan^TM^, GE Healthcare, Chicago, IL, USA). The antibody signal was normalized to the total protein levels; the membranes were stained with Ponceau S and digitalized in a ChemiDoc Imaging Densitometer (Bio-Rad, Hercules, CA, USA). Next, the membranes were washed with TBS-T (TBS buffer with 0.05% Tween-20) to remove the staining. Membranes were then blocked with 5% (*w*/*v*) bovine serum albumin in TBS for 30 min. Finally, each membrane was incubated overnight at 4 °C with the respective primary antibody: anti-MMP2 (rabbit polyclonal antibody, 1:2000 in TBS-T, Abcam ab37150), anti-TIMP1 (rabbit polyclonal antibody, 1:1000 in TBS-T, GeneTex GTX108254), and anti-vWF (rabbit polyclonal antibody, 1:15,000 in TBS-T, Dako A 0082). In any case, the membranes were washed thrice with TBS-T for 10 min and incubated with a fluorescent secondary goat anti-rabbit antibody (1:15,000 in TBS-T, LI-COR IR Dye 800 CW, 926-32211) for 1 h at room temperature. The membranes were rewashed thrice with TBS-T for 10 min before signal acquisition. The detection was carried out with the Odyssey imaging system (LI-COR Biosciences, Lincoln, NE, USA). Blot scans were analyzed using Image Studio^TM^ Lite software (LI-COR Biosciences, Lincoln, NE, USA).

### 4.6. Statistical Analysis

Categorical data are presented as percentages. Continuous demographical and molecular data are presented as median (IQR), except when stated otherwise. Chi-squared and Welch’s tests were applied to detect differences between cases and controls in categorical and continuous variables. The protein quantification data were log2 transformed and normalized for proteome analysis by the z-score. The differences between patients with CAD or AVS and controls were tested with a moderated t-test using the R package limma. For slot blot analysis, data normality was tested by the D’Agostino & Pearson omnibus method. The differences between groups were tested with a one-way ANOVA or a Kruskal-Wallis test, depending on the distribution being normal or non-normal. A post-hoc Tukey or Dunn’s test was used to compare group means/mean ranks, respectively.

The Spearman correlation matrix was computed with the R software (version 4.1.3) using ggcorrplot, corrplot.

In all cases, *p* < 0.05 was considered significant.

## Figures and Tables

**Table 1:** Clinical, biochemical, and demographic characteristics of the patients (CAD/AVS) and the controls of the discovery and testing cohorts.

	Discovery Cohort				Testing Cohort			
	(CAD/AVS + Controls) (N = 23)	CAD/AVS (N = 12)	Controls (N = 11)	*p*-Value	(CAD/AVS + Controls) (N = 29)	CAD/AVS (N = 19)	Controls (N = 10)	*p*-Value
Sex (man)	12 + 11	67%	82%	n.s.	19 + 10	100%	50%	<0.001
Age (years)	12 + 5	70 (65–75)	63 (59–76)	n.s.	19 + 10	78 (74–81)	69 (63–75)	<0.01
Body Mass Index	11 + 5	29 (25–30)	30 (24–35)	n.s.	19 + 10	27 (4)	29 (9)	n.s.
Total cholesterol (mg/dL)	3 + 5	142 (122–194)	161 (107–179)	n.s.	7 + 10	164 (139–189)	142 (129–161)	n.s.
HDL (mg/dL)	3 + 5	35 (34–40)	46 (39–46)	n.s.	7 + 10	55 (40–56)	46 (42–60)	<0.05
LDL (mg/dL)	3 + 5	78 (65–128)	77 (65–101)	n.s.	7 + 10	85 (80–112)	67.5 (58–79)	<0.05
Hemoglobin (g/dL)	12 + 5	14 (13–15)	14 (13–15)	n.s.	19 + 10	14 (13–15)	12 (11–13)	<0.05
Hematocrit (%)	12 + 5	41 (40–44)	42 (37–44)	n.s.	19 + 10	42 (39–43)	37 (33–38)	<0.01
Platelets (thousands/mL)	12 + 5	237 (198–246)	277 (235–280)	n.s.	19 + 10	207 (181–228)	270 (214–280)	<0.01
Creatinine (mg/dL)	12 + 5	0.8 (0.7–1)	0.87 (0.8–1)	n.s.	19 + 10	0.95 (0.9–1.1)	0.22 (0.7–1)	n.s.
Ejection fraction (%)	7 + 5	36 (34–54)	63 (62–65)	<0.001	15 + 9	49 (40–62)	63 (62–64)	<0.001
Hypertension	12 + 11	83%	45%	n.s.	19 + 10	95%	90%	n.s.
Dyslipidemia	12 + 11	75%	45%	n.s.	19 + 10	79%	100%	n.s.
Ex-smoker or smoker	12 + 5	50%	60%	n.s.	19 + 10	68%	40%	n.s.
Diabetes mellitus	12 + 11	42%	27%	n.s.	19 + 10	58%	60%	n.s.
Angina	10 + 11	50%	0%	n.s.	19 + 10	74%	0%	n.s.

Data are presented as % or as median (IQR)*;* CAD, coronary artery disease; AVS, aortic valve stenosis; HDL, High-Density Lipoprotein; LDL, Low-Density Lipoprotein; n.s., non-significant. Welch’s test and Chi-squared test were used for continuous and categorical variables, respectively.

## Data Availability

All relevant data have been deposited to the ProteomeXchange Consortium via the PRIDE (http://www.ebi.ac.uk/pride, (accessed on 20 October 2022)) partner repository with the data set identifier PXD036800.

## Supplementary Materials

The following are available online at https://www.mdpi.com/article/10.3390/ijms232113579/s1, Table S1: Proteome label-free quantification data.
